# *NnARF17* and *NnARF18* from lotus promote root formation and modulate stress tolerance in transgenic *Arabidopsis thaliana*

**DOI:** 10.1186/s12870-024-04852-9

**Published:** 2024-03-02

**Authors:** Cheng Libao, Liang Shiting, Zhao Chen, Li Shuyan

**Affiliations:** 1 College of Horticulture and Landscape Architecture, Yangzhou University, Yangzhou, Jiangsu P. R. China; 2 College of Guangling, Yangzhou University, Yangzhou, Jiangsu P. R. China

**Keywords:** lotus, Adventitious root, *NnARF17*, *NnARF18*, *Arabidopsis*

## Abstract

Auxin response factors (ARFs) play a crucial role in regulating gene expression within the auxin signal transduction pathway, particularly during adventitious root (AR) formation. In this investigation, we identified full-length sequences for *ARF17* and *ARF18*, encompassing 1,800 and 2,055 bp, encoding 599 and 684 amino acid residues, respectively. Despite exhibiting low sequence homology, the *ARF17-* and A*RF18-*encoded proteins displayed significant structural similarity and shared identical motifs. Phylogenetic analysis revealed close relationships between *NnARF17* and *VvARF17*, as well as *NnARF18* and BvARF18. Both *ARF17* and A*RF18* demonstrated responsiveness to exogenous indole-3-acetic acid (IAA), ethephon, and sucrose, exhibiting organ-specific expression patterns. Beyond their role in promoting root development, these ARFs enhanced stem growth and conferred drought tolerance while mitigating waterlogging stress in transgenic *Arabidopsis* plants. RNA sequencing data indicated upregulation of 51 and 75 genes in *ARF17* and *ARF18* transgenic plants, respectively, including five and three genes associated with hormone metabolism and responses. Further analysis of transgenic plants revealed a significant decrease in IAA content, accompanied by a marked increase in abscisic acid content under normal growth conditions. Additionally, lotus seedlings treated with IAA exhibited elevated levels of polyphenol oxidase, IAA oxidase, and peroxidase. The consistent modulation of IAA content in both lotus and transgenic plants highlights the pivotal role of IAA in AR formation in lotus seedlings.

**Supplementary Information:**

The online version contains supplementary material available at 10.1186/s12870-024-04852-9.

## Key message

Indole-3-acetic acid significantly influences the formation of short-borne roots in lotus seedlings. The overexpression of two auxin-responsive genes, *NnARF17* and *NnARF18*, in *Arabidopsis* resulted in enhanced root development.

**Supplementary Information:**

The online version contains supplementary material available at 10.1186/s12870-024-04852-9.

## Introduction

Lotus root (*Nelumbo nucifera* Gaertn) is a member of the Nymphaeaceae family, which encompasses only one other species: *Nelumbo lutea* [1, 2]. It serves as a significant off-season vegetable [3] and has one of the largest aquatic vegetable production areas in China, particularly in the southern regions of the Yangtze River Basin, including Hubei, Hunan, Jiangsu, Guangdong, Guangxi, and Jiangxi. These areas provide optimal climatic conditions for lotus root growth, generating substantial profits for local farmers. Beyond its role as a vegetable, lotus is utilized in various products, such as drinks, tea, salt lotus, and glutinous rice lotus, which have gained international popularity [4]. Due to its rich composition of special substances, lotus root is recognized as a functional food or medicine.

The short-borne roots (adventitious roots (ARs)) of lotus play a crucial role in plant growth and the formation of storage organs, compensating for the underdevelopment of the primary root. Any factor promoting the development of ARs contributes to enhanced plant metabolism and increased yield of storage organs. Root systems in plants exhibit diverse morphologies influenced by genetic and environmental factors. Plant roots are categorized into tap roots (primary roots), basal roots, ARs, and lateral roots based on their occurrence [5]. Lotus plants, in particular, may exhibit severe degeneration of the main root, leading to the production of numerous ARs in the hypocotyl of seedlings or the internodes of storage organs to sustain normal growth. The ARs are considered secondary root systems, and their formation involves three biological stages: induction, initiation, and expression [6, 7]. The initial stage involves the transition of cell function, where normal cells differentiate into meristematic cells capable of developing into ARs. The second stage is the primordial establishment, during which meristematic cells differentiate into primordial ARs [8]. In the final stage, the AR primordium continues to develop, leading to the breakout of the stem or leaf epidermis, resulting in fully formed ARs [9]. This entire developmental process is tightly regulated by multiple factors, including genetic elements (gene regulators, and microRNAs) and environmental conditions [8]. Consequently, AR formation is considered a heritable quantitative trait [10].

Indole-3-acetic acid (IAA) stands as a pivotal plant hormone crucial for regulating various facets of plant growth and development. Serving as an essential regulator, it plays a fundamental role in cell division, elongation, and differentiation [11, 12]. Additionally, IAA collaborates with other factors to influence organ formation, playing a key role in processes such as primordial root induction, bud differentiation, leaf development, flowering, and fruit formation [13, 14]. In particular, auxin, a type of IAA, triggers the development of the root cap, promotes root formation during the initiation stage, and facilitates the occurrence of ARs [15, 16], with a close correlation to auxin transport [17, 18]. Reports indicate that increased endogenous IAA levels or decreased IAA oxidase (IAAO) activity accelerate AR development [19, 20]. Exogenous IAA significantly influences AR formation by promoting cell division and primordium formation [11, 12]. Notably, the role of IAA in lotus AR formation is dose-dependent, with low concentrations dramatically accelerating AR development and high concentrations exhibiting the opposite effect [21]. The impact of abscisic acid (ABA) on AR formation is both dose- and species-specific. Generally, ABA signaling negatively affects AR development [22]. Further investigations reveal that ABA primarily inhibits AR formation during the induction stage of the primordium and the root elongation stage in softwood cuttings [23]. In the case of rice, ABA inhibits the rate of AR growth by influencing gibberellin A (GA) biosynthesis and IAA signaling [24]. The ABA / (IAA + GA3) ratio serves as an indicator of rooting capacity [25].

Auxin response factors (ARFs) play a crucial role in various plant physiological processes, encompassing the development of leaves, flowers, and roots, as well as processes like maturation, senescence, fruit abscission, and stress response. ARFs actively participate in the development of roots, including primary, lateral, and ARs, in plants. In *Arabidopsis*, *AtARF2* is recognized as a pivotal factor regulating root tip cell division, thereby influencing the formation of primary roots [26]. Additionally, *atARF10*/*atARF16* plays a role in root development by influencing root cap formation [27]. This phenomenon is also observed in rice, where the role of *OsARF1* is closely associated with root cap development [28]. In *OsARF12*-mutant rice, there is a decrease in root expansion, leading to a significant reduction in the number of primary roots [29]. Transgenic plants with constitutive expression of *atARF7* exhibit enhanced lateral root formation compared to wild-type plants [30]. Further analysis reveals that *AtARF7* interacts with MYB transcription factors to regulate lateral roots [31]. It is noteworthy that the roles of ARFs can vary among species. For instance, the overexpression of *ARF17* in *Arabidopsis* results in a significant decrease in the number of ARs [32], while *PeARF17* promotes the formation of ARs in transgenic plants [33].

Previously, our research revealed the significant influence of IAA on the formation of ARs in lotus seedlings [34]. Building on this, we identified *NnARF17* and *NnARF18* as key players in AR development. Given their association with the IAA signal transduction pathway, we hypothesized that *NnARF17* and *NnARF18* could serve as crucial regulators in the process of AR formation in lotus. This study aimed to provide a detailed analysis of the isolation of *NnARF17* and *NnARF18*, their expression in lotus, and their primary functions in transgenic *Arabidopsis* plants. Additionally, we explored the potential pathways involved in root formation in these transgenic plants. The insights gained from this investigation contribute to a better understanding of the role of IAA signaling and establish a necessary foundation for further exploration of the regulatory networks governing AR formation in lotus seedlings.

## Materials and methods

### Preparation of plant materials

For our experiments, Taikong Lotus 36, a seed lotus variety widely cultivated in China, was chosen due to its abundant seed production. This lotus variety, developed by The Guangchang Bailian Institute, was procured from a market and sown in an experimental field of aquatic vegetables at Yangzhou University, Southeast China. Throughout the growth season (April to October), the field maintained a water depth of 20–25 cm from April to June, increased to approximately 40–50 cm in the summer months (July to August), and then returned to 20–30 cm in autumn (September to October). The average temperature was kept within the range of 25–35 °C during the day and 18–25 °C at night to support optimal plant growth. After harvesting, the seeds were stored at about 10–20 °C in a glass container.

### The influence of exogenous IAA on ARs formation in lotus seedlings

To initiate the germination process, the lotus seed coat was ruptured, and the seeds were immersed in water at 26 °C in darkness for approximately 6 days. Fifty seedlings, each with two leaves, were selected and treated with 10 µM IAA for 2 days, after which they were transferred into water for continuous cultivation. The abundance of ARs was observed at 0, 2, 4, 6, and 8 days, with ARs measuring ≥ 0.2 cm in length from the epidermis being used for counting. Control plants, treated with water, were included in the experiment for comparison, and the entire process was conducted in triplicate.

Moreover, hypocotyls from both the treated and control plants were chosen for microstructural analysis at six different time points. The hypocotyls were sectioned into pieces measuring 3 × 3 × 2 mm (length × width × height) and then placed in a glass container filled with fixing fluid (free fatty acids, more than 20 times the volume of the samples). The container, containing the fixed sample, underwent a vacuum process using a syringe for approximately 5 s, followed by a 5 min opening. This cycle was repeated three times, and the containers were then transferred to room temperature for approximately 12–24 h. Subsequently, the samples underwent dehydration using ethanol at concentrations of 50%, 70%, 85%, 95%, and absolute for 30 min each. Following dehydration, a mixture of absolute ethanol and pure xylene (1:1, v/v) was applied, followed by pure xylene for 30 min. Paraffin debris was used to fill the container containing the samples, and it was placed on a platform at room temperature for at least 12 h to prepare paraffin blocks. The paraffin blocks were sliced using a microtome to produce wax tape (10 microns in thickness), which was then transferred to a glass slide. The wax tape underwent sequential treatments of pure xylene, a mixed solution of xylene and absolute ethanol (1:1), and absolute ethanol, each for 5–10 min. Finally, the microstructure of the developed ARs was visualized using an optical microscope after the slide was air-dried.

### Cloning of *NnARF17* and *NnARF18*

The expression levels of *NnARF17* and *NnARF18* during AR formation in lotus seedlings, as revealed by transcriptome data [35], prompted the cloning of these genes. The full-length sequences of *NnARF17* and *NnARF18* were sourced from the National Center for Biotechnology Information (NCBI) database. Gene-specific primers were designed using Primer 5.0 software. *NnARF17*: forward primer, 5’-ATGGCGTTGCAGAGGGTGAGT-3’; reverse primer, 5’-TAAGGAACAAGCTTCCACTGCTGAG-3’. *NnARF18*: forward primer, 5’-ATGGCGTATGGAGATAGCTG-3’; reverse primer, 5’-TATCTCAGTCTTTAGGTCTGAATCC-3’. Total RNA was extracted from lotus leaves using the RNeasy MinElute Cleanup Kit (QIAGEN, Hilden, Germany), following the manufacturer’s instructions. DNA contamination was eliminated through DNAase I treatment before synthesizing the first cDNA strand. The PCR system included 2.5 µL of dNTPs, 2 µL of each primer (forward and reverse), 2.5 µL of MgCl_2_, 2 µL of Taq polymerase (5 U), 2 µL of cDNA fragments, and 7 µL of dH_2_O in a total reaction volume of 20 µL. The PCR program consisted of pre-denaturation at 94 °C for 1 min; 35 cycles of denaturation at 94 °C for 1 min, annealing at 56–60 °C for 1 min, and extension at 72 °C for 1 min; and a final extension at 72 °C for 10 min. The target gene fragments were purified using the GeneJET Gel Extraction Kit (Thermo Fisher Scientific, Waltham, MA, USA) and inserted into a cloning vector (pMD 18-T vector; TaKaRa, Kusatsu, Japan). Recombination of these gene fragments was achieved using *DH5α*, and the resulting sequences were verified by Sangon Biotechnology Co., Ltd. (Shanghai, China).

### Sequence analysis of *NnARF17* and *NnARF18*

For the comparative analysis of *NnARF17* and *NnARF18* sequences, DNAman (https://www.onlinedown.net/article/10009213.htm) was employed. To identify conserved domains, the Simple Modular Architecture Research Tool software program was utilized. The construction of phylogenetic trees for *NnARF17* and *NnARF18* was accomplished using DNAman and MEGA X software.

Analysis of conserved motifs in *NnARF17* and *NnARF18* was performed using DNAman and the online software program MEME server v5.4.1 (http://meme-suite.org/tools/meme). The sequences of *NnARF17* and *NnARF18* were standardized and then submitted to the MEME software. In the system options, “zoops” and “ten” motifs in the required box were set according to the instructions. Finally, TBtools software was utilized to visualize the output.

### Expression profiling analysis of *NnARF17* and *NnARF18*

The quantitative reverse transcription polymerase chain reaction (qRT-PCR) technique was employed to assess the expression levels of *NnARF17* and *NnARF18* in lotus seedlings subjected to treatments with 10 µM IAA, 20 g/L sucrose, and 200 mg/L ethephon. Plant samples treated at five time-points (0, 2, 4, 6, and 8 days) were selected, and total plant RNA was extracted using the RNeasy MinElute Cleanup Kit (QIAGEN).

Gene expression was analyzed in various plant organs, including ARs, leaves, stems, flowers, and fruits. Genomic DNA contamination was removed using DNase I, and first-strand cDNA was synthesized with 3 µg of purified RNA utilizing the RevertAid First Strand cDNA Synthesis Kit (Fermentas, Waltham, MA, USA). The following primers were used. *NnARF17*: forward primer: 5′-TCTGTGCAGAATCGTGTCGGTTATG-3′, reverse primer: 5′-GAACTTACTCCACCCAGTCGTCAAC-3′; *NnARF18*: forward primer, 5′-ATGAGCCGACGAGTCCTGATCC-3′, reverse primer, 5′-CCGTGGTTGACCTCTGAAGATGTG-3′. β-Actin served as the internal standard with the following primers: forward primer, 5′-AACCTCCTCCTCATCGTACT-3′, reverse primer 5′-GACAGCATCAGCCATGTTCA-3′. qRT-PCR analysis was conducted using SuperReal PreMix Plus (Tiangen, China) on an Mx 3000P machine (STRATAGENE, Santa Clara, CA, USA). The PCR reaction included a 25 µL mixture with 12.5 µL of SYBR Premix Ex Taq II (Tli RNaseH Plus) (2×), 1 µL of each primer, 3 µL of cDNA, and 8.5 µL of dH_2_O. The PCR program comprised 40 cycles of 94 °C for 30 s, 95 °C for 5 s, and 60 °C for 60 s. mRNA levels were determined using the 2^–△△Ct^ method, and all gene expression experiments were performed in triplicate.

### Vectors construction of *NnARF17* and *NnARF18*

*NnARF17* and *NnARF18* were integrated into the pGEM-T vector and introduced into *Escherichia coli* for replication. Utilizing *Bam*HI and *Kpn*I enzymes, the recombinant forms of these genes were digested to isolate the targeted genes with specific restriction enzyme sites. The plant transformation vector pSN1301, featuring a CaMV 35 S promoter, was selected to create pSN1301::*NnARF17* and pSN1301::*NnARF18*, subsequently transferred into *Agrobacterium tumefaciens* strain GV3101 for *Arabidopsis* plant preparation. The floral dip method, as described by Clough et al. [36], was employed in this experiment.

### Generation of transgenic *Arabidopsis* plants

Seeds of the T0 generation were initially screened on Murashige and Skoog (MS) medium supplemented with 20 µg/g hygromycin B to identify “positive” plants. Subsequently, these plants were transferred to a greenhouse for continuous cultivation at 22 °C (12 h/light and 12 h/dark). qRT-PCR was conducted to further confirm the identity of “positive” plants during the cultivation period, using the same PCR mixture and program as employed for gene cloning.

Functional analysis of *NnARF17* and *NnARF18* on root formation.

Gene functionality was assessed at the six-leaf stage in transgenic *Arabidopsis* plants, with wild-type plants serving as controls.To explore their impact on root formation and plant growth, both transgenic and wild-type plants were subjected to sterilization with 80% alcohol for 20 s, followed by a 10% sodium hypochlorite treatment for 20 min. Sterilized seeds from both plant types were then placed on MS medium and a base material composed of soil and vermiculite in a 1:1 ratio (v/v).

### Subcellular localization of *NnARF17* and *NnARF18* in tobacco plants

Tobacco (*Nicotiana tabacum* L.) seeds underwent initial sterilization before being sown in soil for germination at 26 °C in the dark. Upon reaching the eight-leaf stage, seedlings were transferred to an illuminating incubator, alternating between 26 °C for 12 h in the light and 22 °C for 12 h in the dark.

Recombinant plasmids of *NnARF17*, *NnARF18* and *pCAMBIA1300-35 S-EGFP* (expressing vector) were digested by *Bam*HI and *Xba*l enzymes for about 2 h in a water bath pot under 37 °C condition. DNA gel recovery kit (TaKaRa, Kusatsu, Japan) was used to obtain fragments of these two genes and expressing vector according to instruction of the Kit. *NnARF17* and *NnARF18* were inserted into pCAMBIA1300-35 S-EGFP by T4 DNA ligase at 16 °C for 12 h. Recombinant plansmids of *NnARF17*, *NnARF18* were identified by PCR method and double enzymes’ digestion.

*pCAMBIA1300-35 S-EGFP*::*NnARF17* and *pCAMBIA1300-35 S-EGFP*::*NnARF18* were introduced into *A. tumefaciens* (GV3101) through electroconversion, followed by a 2-day incubation at 30 °C. *A. tumefaciens* was harvested from the solid medium, suspended in 10 mL of YEB liquid medium, and incubated at 170 rpm/min for 1 h. After centrifugation at 4,000 rpm for 4 min, the supernatant was discarded, and the precipitate was resuspended in 10 mM MgCl_2_ to achieve an OD_600_ of 0.6. Using a 1 mL syringe without a nozzle, the lower epidermis of tobacco leaves was injected. The injected plants were cultured under low-intensity light for 2 days. Labeled leaves were placed on glass slides and observed under a laser confocal microscope.

### Analysis of RNA sequencing data

#### RNA sequencing analysis for *NnARF17* and *NnARF18*

The seeds of both transgenic and wild-type plants underwent sterilization before germinating on base materials at 26 °C. Subsequently, germinated seeds were transferred to a greenhouse for continuous cultivation, maintaining an average temperature of 23 ± 2 °C under 12 h/light and 12 h/dark conditions.

For genome-wide gene expression analysis, RNA sequencing (RNA-seq) was conducted when the seedlings reached the six-leaf stage. Approximately 2–3 µg of total plant RNA from both transgenic and wild-type plants was extracted for constructing libraries (control library, *NnARF17* library, and *NnARF18* library). Library construction followed the procedure described by Cheng et al. [35], and the libraries were sequenced by Nanjing Jisi Huiyuan Biotechnology Co., Ltd. (Nanjing, China).

#### Annotation of differentially expressed genes

The genome-wide gene expression data from each library underwent a parallel comparison using Illumina sequencing platforms. Subsequently, differentially expressed genes (DEGs) were identified using the NOISeq method, as outlined by Cheng et al. [21]. A fold change in expression ≥ 2 and a divergence probability ≥ 0.8 were set as the thresholds for DEG identification.

To elucidate the functional aspects of these DEGs, gene annotation was performed using the Gene Ontology (GO) tool. DEGs were categorized into three ontologies–molecular function, cellular components, and biological processes–based on the instructions provided by the GO tool. Enrichment analysis was conducted by comparing DEGs with the *Arabidopsis* genome obtained from the NCBI database, utilizing a hypergeometric test to determine significantly enriched GO terms. Furthermore, all DEGs were classified into various biological pathways through enrichment analysis, comparing them with the NCBI database genome, and utilizing the Kyoto Encyclopedia of Genes and Genomes.

#### Determination of IAA, ABA, GA3, peroxidase, polyphenol oxidase, and IAAO content

The seeds of transgenic (*NnARF17* and *NnARF18*) and wild-type *Arabidopsis* plants were sown on base material and placed in an illuminating incubator at 22 °C. The determination of IAA, ABA, GA3, and peroxidase (POD) contents occurred when the plants reached the six-leaf stage. Additionally, lotus seed coats were broken and subjected to water for germination at 26 °C. Seedlings treated with 10 µM IAA for 2 days were then transferred into water for continuous cultivation. Five time points (0, 2, 4, 6, and 8 days) were selected for the analysis of IAA, GA3, ABA, POD, polyphenol oxidase (PPO), and IAAO contents.

Samples were initially flash-frozen in liquid nitrogen and ground into powder using a rod. Approximately 3 g of powder from transgenic and wild-type plants were utilized for the extraction of IAA, ABA, and GA3. In a centrifuge tube, 600 µL of a reagent composed of isopropyl alcohol, water, and concentrated hydrochloric acid (2:1:0.002, v/v/v) was combined with the sample and agitated at 4 °C for 30 min. The resulting mixture underwent centrifugation at 12,000 rpm at 4 °C for 10 min. The supernatant was discarded, and the precipitate was collected, dried with nitrogen, and dissolved in filtered methanol (50 mL). The solution was then analyzed for IAA and ABA contents using a liquid chromatograph (Sigma, Shanghai, China) [37, 38]. The GA3 content was determined following the method outlined by Nhujak et al. [39]. For the determination of POD, PPO, and IAAO contents, 0.1 g of freeze-dried sample was homogenized in 1 mL of extraction solution in an ice bath. After centrifugation at 8,000 rpm at 4 °C for 10 min, the supernatant was used for subsequent experiments. Each sample was added to a 1 mL glass colorimetric dish, and absorbance values at 470, 410, and 530 nm were measured to quantify POD, PPO, and IAAO, respectively. These experiments were conducted in triplicate.

#### Stress tolerance of transgenic and wild-type plants

Approximately 100 seeds of both transgenic and wild-type plants were soaked in water for 48 h and then transferred to a medium (v/v, soil: vermiculite = 1:1) at 22 °C. Seedlings at the six-leaf stage were employed to evaluate waterlogging, drought, and salt tolerance. For waterlogging analysis, transgenic and wild-type plants were placed in a tray with water at a depth of 2 cm for approximately 20 days, followed by transfer to normal growth conditions. Measurements of fresh weight, dry weight, and stem growth were taken after 10 days of cultivation.To assess drought tolerance, both types of plants were initially grown under identical water management conditions, and then water was withheld for about 10 days. The survival rates of the plants were determined after 7 days of water recovery. For salt tolerance assessment, transgenic and wild-type plants were treated with 100 mM NaCl, and the survival rates were evaluated after approximately 10 days. Three biological replicates were utilized for each type of plant, and each replicate involved at least 50 seedlings.

### Statistical analysis

Statistical analyses were conducted using Origin Pro software (version 8.0; Origin Inc., Framingham, MA, USA). Each experiment was carried out in triplicate, and the means ± standard errors of the three repetitions were calculated. All figures display mean values with standard errors. Student’s t-tests were employed to determine significant differences, with differences considered significant at *p* < 0.05.

## Results

### IAA influence on lotus AR development

**Fig. 1:**
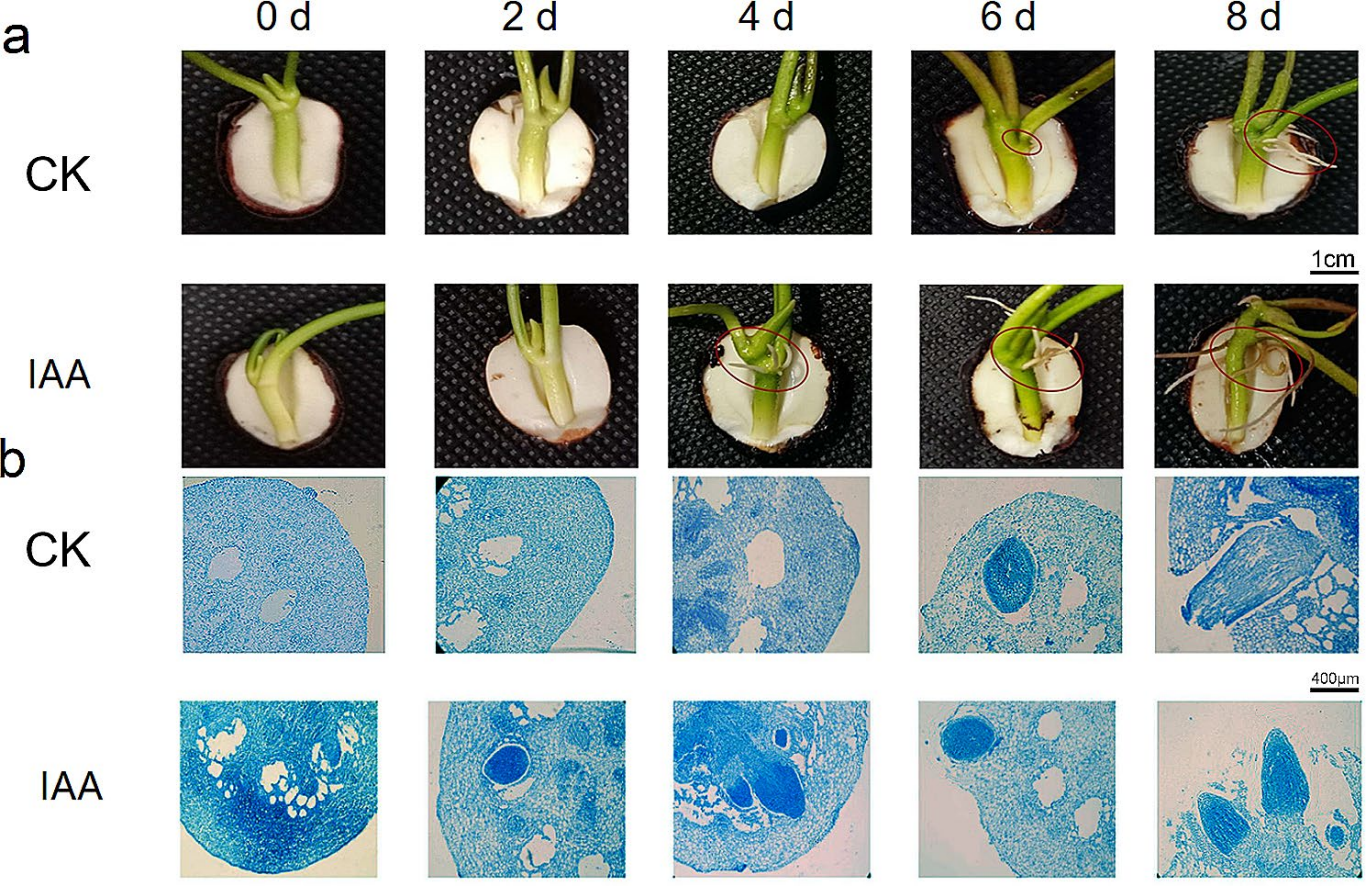
The role of exogenous application of IAA on ARs formation in lotus seedlings. **a**. Observation of root growth from morphology of seedlings treated with 10 µM IAA for 0 d, 2 d, 4 d, 6 d, and 8 d. Analysis of microstructure on ARs formation at the hypocotyls of lotus seedlings treated with 10 µM IAA for 0 d, 2 d, 4 d, 6 d, and 8 d. Ellipses represents ARs or ARs primordium

To assess the impact of IAA on lotus AR formation, seedlings underwent a 2-day treatment with 10 µM IAA. The results demonstrated that IAA treatment significantly enhanced AR development, with ARs breaking through the hypocotyl epidermis after 4 days, while control plants required 6 days for AR appearance (Fig. 1a). Concurrently, microstructural analysis revealed clear induction and development of AR primordia after 2 days, with visible ARs in the hypocotyl after 4 days in treated seedlings (Fig. 1b). In contrast, control plants exhibited a longer duration for complete AR formation. These findings indicate that IAA expedites the AR formation process, particularly during the induction stage in lotus seedling hypocotyls.

### Cloning and sequences analysis of *NnARF17* and *NnARF18*

**Fig. 2:**
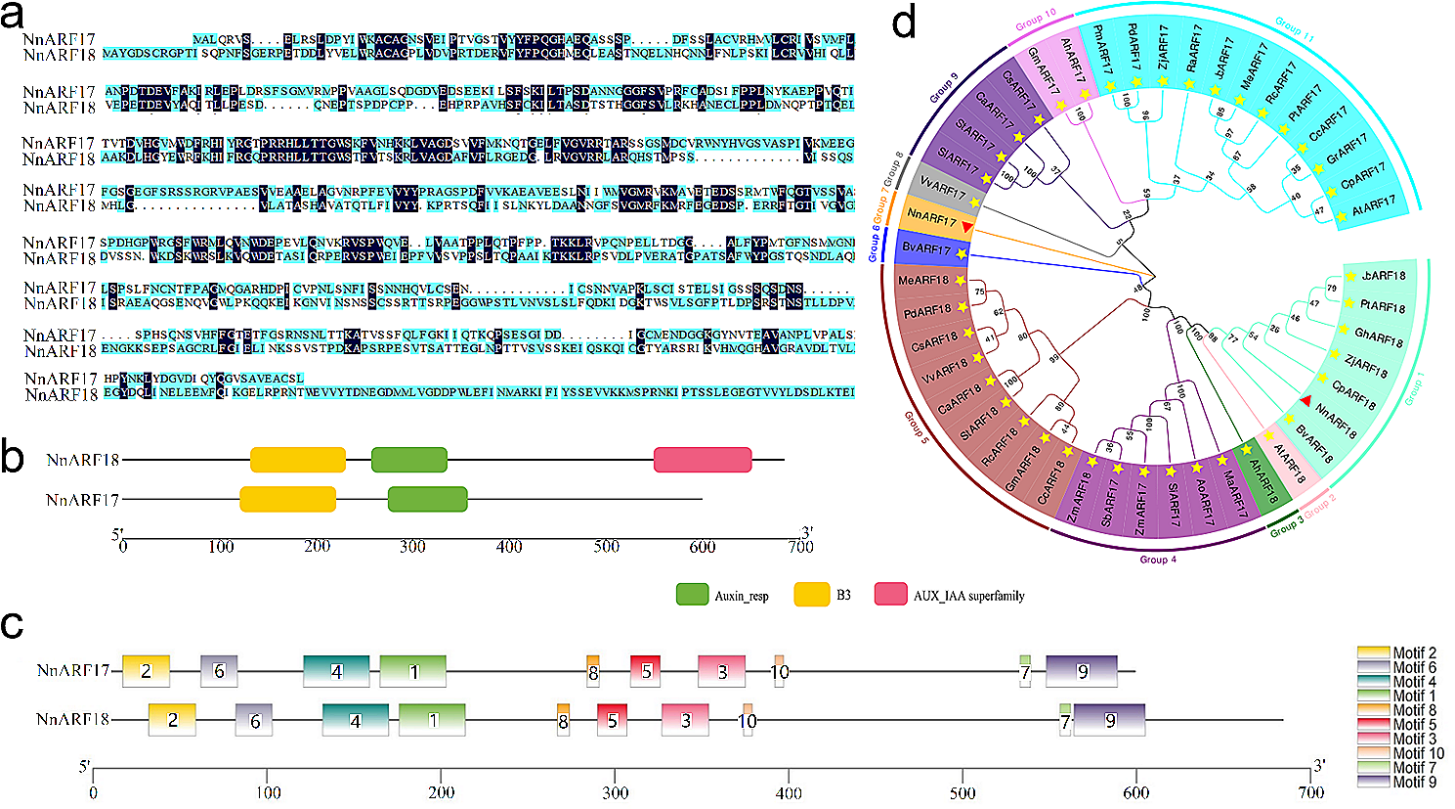
Comparison and phylogenetic tree analysis of *NnARF17* and *NnARF18*. **(a)** Comparison of *NnARF17* and *NnARF18* with amino acid sequences. **(b)** Domain analysis of *NnARF17* and *NnARF18* encoded proteins, and the box different color represents conserved region. **(c)** Motifs analysis of *NnARF17* and *NnARF18* encoded proteins. Boxes of different colors represent the ten putative motifs, and the boxes with the same color represent the same motif in structure of these three genes. **(d)** Phylogenetic tree analysis of *NnARF17* and *NnARF18* encoded proteins with *ARF17* and *ARF18* encoded proteinof other species, and total eleven groups with different color were detected. The red triangle represents the positions of the *NnARF17* and *NnARF18* encoded proteins in the phylogenetic tree

In a prior investigation, we observed the induction of two sucrose-responsive auxin genes significantly promoting AR formation in lotus seedlings. Subsequently, these genes were cloned using RT-PCR, resulting in open reading frames of 1,797 and 2,052 bp for NnARF17 and NnARF18, respectively. These encoded proteins of 599 and 684 amino acid residues (Additional Table 1; Fig. 2a). Comparative analysis with data from the NCBI database revealed the presence of conservative domains in *ARF17* and *ARF18* encoding proteins in *Camellia*, *Macadamia*, *Theobroma*, and *Prunus* (Additional Figs. 1 and 2), leading to their designation as *NnARF17* and *NnARF18*. Despite having low sequence similarity, both *NnARF17* and *NnARF18* contained identical auxin response and B3 homeobox elements (Fig. 2a and b). Furthermore, examination of protein domains indicated their similarity, hinting at potential shared biological functions (Fig. 2c). Phylogenetic analysis identified 11 groups, with *NnARF17* forming an independent group (group 6), highlighting its distant relationship with members of other groups. In contrast, *NnARF18* showed close associations with *BvARF18*, *CpARF18*, *ZjARF18*, *GhARF18*, *PtARF18*, and *JcARF18*, placing them in a shared group (Fig. 2d).

**Table 1:** Expression changes of genes involved in IAA metabolism, response and root formation in transgenic *NnARF17*, *NnARF18* and wild type *Arabidipsis* plants

ID	*NnARF17*	*NnAFR18*		Function annotation
**Genes invovled in hormone metabolism and signal transduction pathway**				
AT1G15520	1.13	------		Pleiotropic drug resistance 12
AT1G32630	-1.32	------		Transcription factor MYC2
AT2G44840	-1.76	------		Ethylene-responsive element binding factor 13
AT4G03585	------	4.11		Cytochrome P450
AT2G20880	------	2.32		Ethylene-responsive transcription factor ERF053
AT2G36270	------	-1.01		bZIP transcription factor family protein
**Genes invovled in root foramtion**				
AT1G15000	9.54	------		Serine carboxypeptidase-like 50
AT4G38860	1.01	------		SAUR-like auxin-responsive protein family
AT4G27260	-1.21	------		Auxin-responsive GH3 family protein
AT3G62090	-2.69	------		Phytochrome interacting factor 3-like 2
AT3G18710	-1.59	------		Plant U-box 29
AT5G10250	------	1.28		Phototropic-responsive NPH3 family protein
AT2G34060	------	1.11		Putative peroxidase

Note: “-----” represented no changes of expression

### qRT-PCR analysis in *NnARF17* and *NnARF18*

**Fig. 3:**
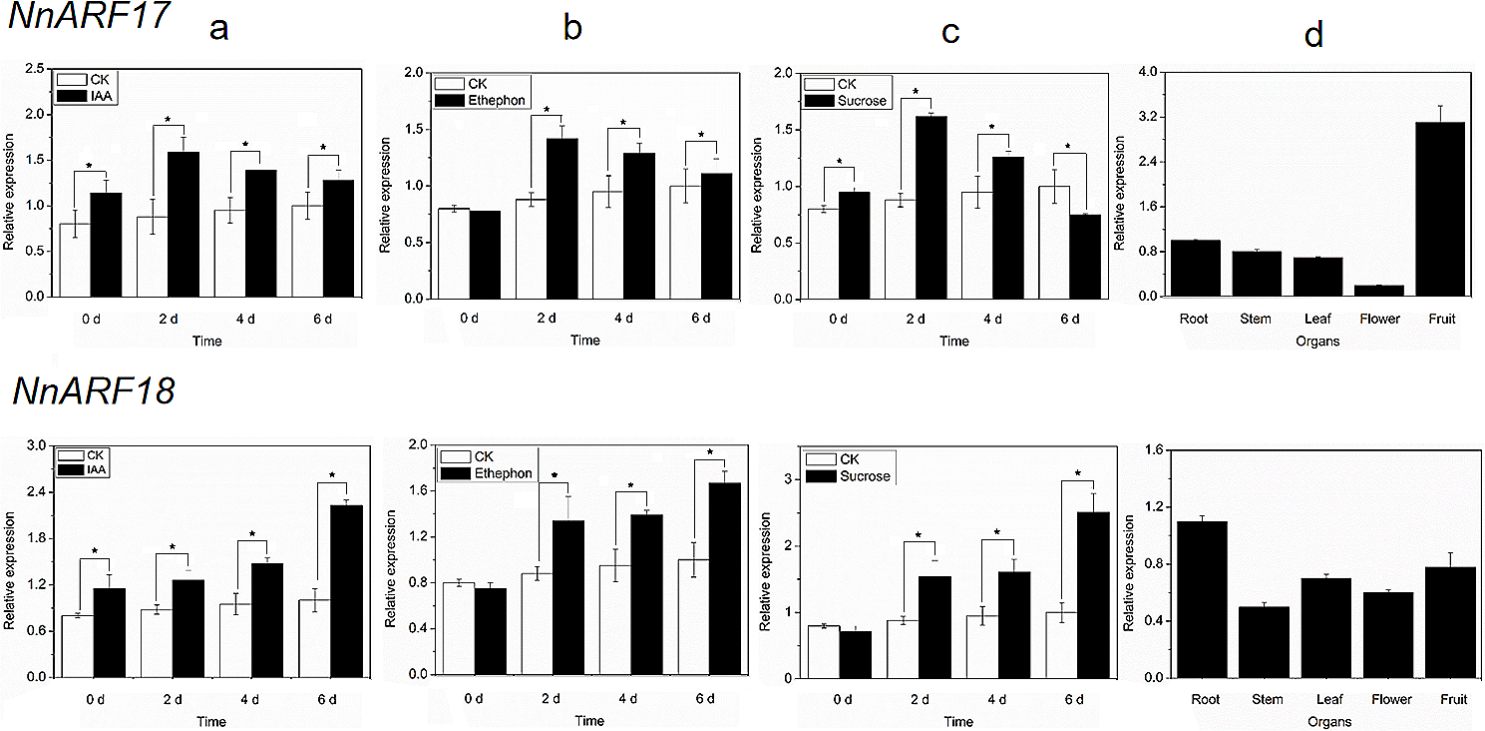
Expression patterns of *ARF17* and *ARF18* with different treatments and in different organs, as determined by qRT-PCR. **(a)** Expression analysis of *NnARF17* and *NnARF18* after IAA treatment. **(b)** Identification of *NnARF17* and *NnARF18* expression in lotus seedlings treated with ethephon. **(c)** Determination of *NnARF17* and *NnARF18* expression in lotus seedlings treated with sucrose. **(d)** Organ-specific expression analysis in roots, stems, leaves, flowers and fruit of lotus plants. The data were recorded as means ± SEs of three biological replicates with about five seedlings in each experiment. Significant differences were carried out and determined by presented as * *p* < *0.05*

The transcriptional profiles of *NnARF17* and *NnARF18* were scrutinized through qRT-PCR, providing consistent results with those obtained through alternative methods. *NnARF17* exhibited similar expression patterns in seedlings treated with IAA, ethephon, and sucrose, with mRNA levels peaking after 2 days of water cultivation. In contrast, *NnARF18* though induced by IAA, ethephon, and sucrose, displayed a distinct expression trend compared to *NnARF17*. Tissue-specific expression patterns of *NnARF17* and *NnARF18* were investigated across various organs, revealing distinctive preferences. The highest transcription levels were observed in fruits, followed by ARs, stems, leaves, and flowers. Notably, *NnARF18* exhibited higher expression in ARs compared to other organs (Fig. 3).

### Subcellular localization of *NnARF17* and *NnARF18*

**Fig. 4:**
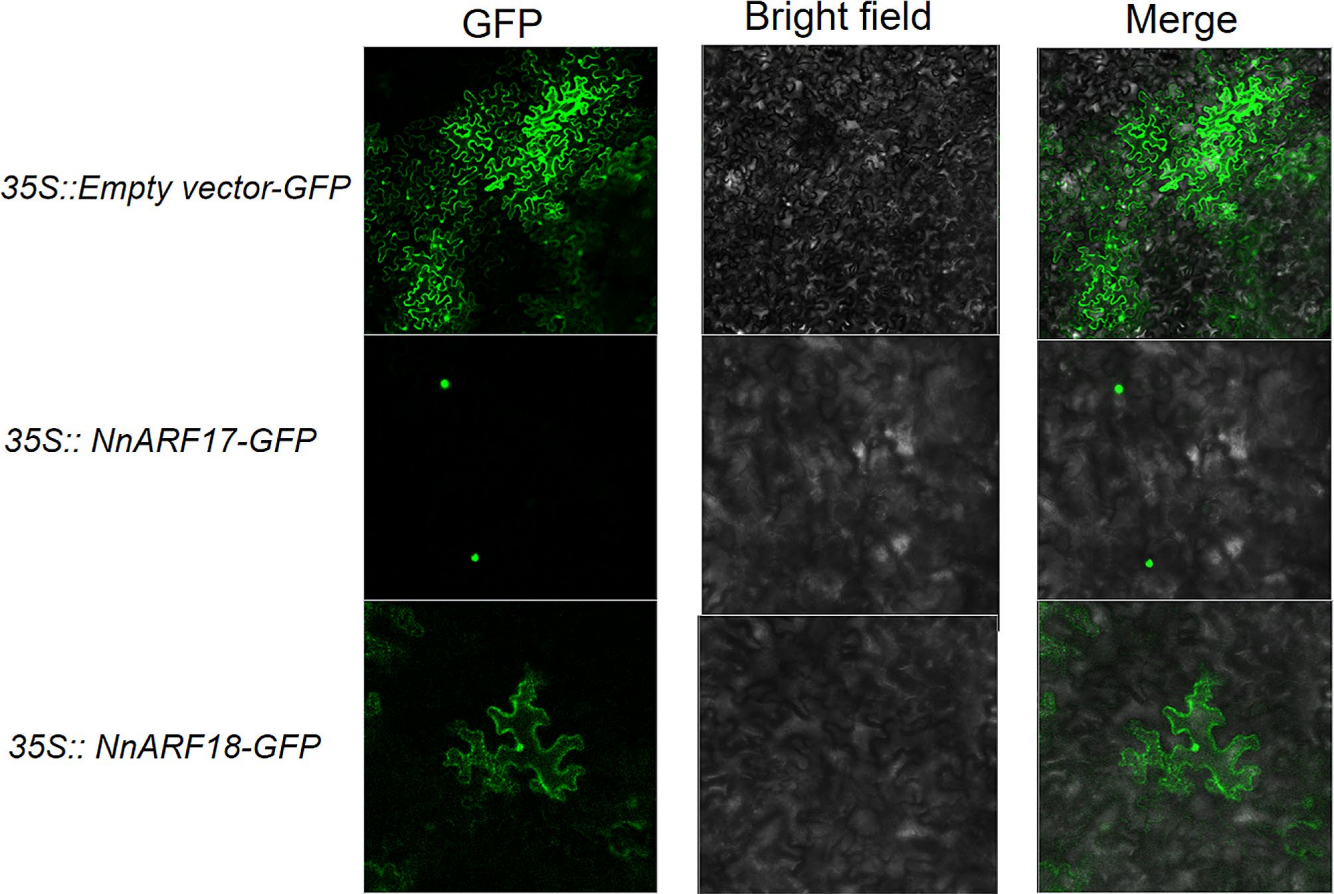
Subcellular localization of *NnARF17 and NnARF18* in tobacco plants

In-depth subcellular localization assays were executed to precisely determine the functional gene locations of *NnARF17* and *NnARF18* within tobacco organelles. The instantaneous transfer of *NnARF17* and *NnARF18* to tobacco plants at the eight-leaf stage was performed, with *35 S::empty vector GFP* serving as the control. The fluorescence signals for GFP were notably robust and evident in the merged field, displaying clear contours in the bright field. *NnARF17* and *NnARF18* exhibited similar localization patterns within tobacco plants, with both genes primarily situated in the nuclei based on their positions in plant cells(Fig. 4). This localization strongly suggests that *NnARF17* and *NnARF18* function as nuclear genes, influencing root formation in transgenic *Arabidopsis* plants.

### Root development in transgenic *Arabidopsis* plants

**Fig. 5:**
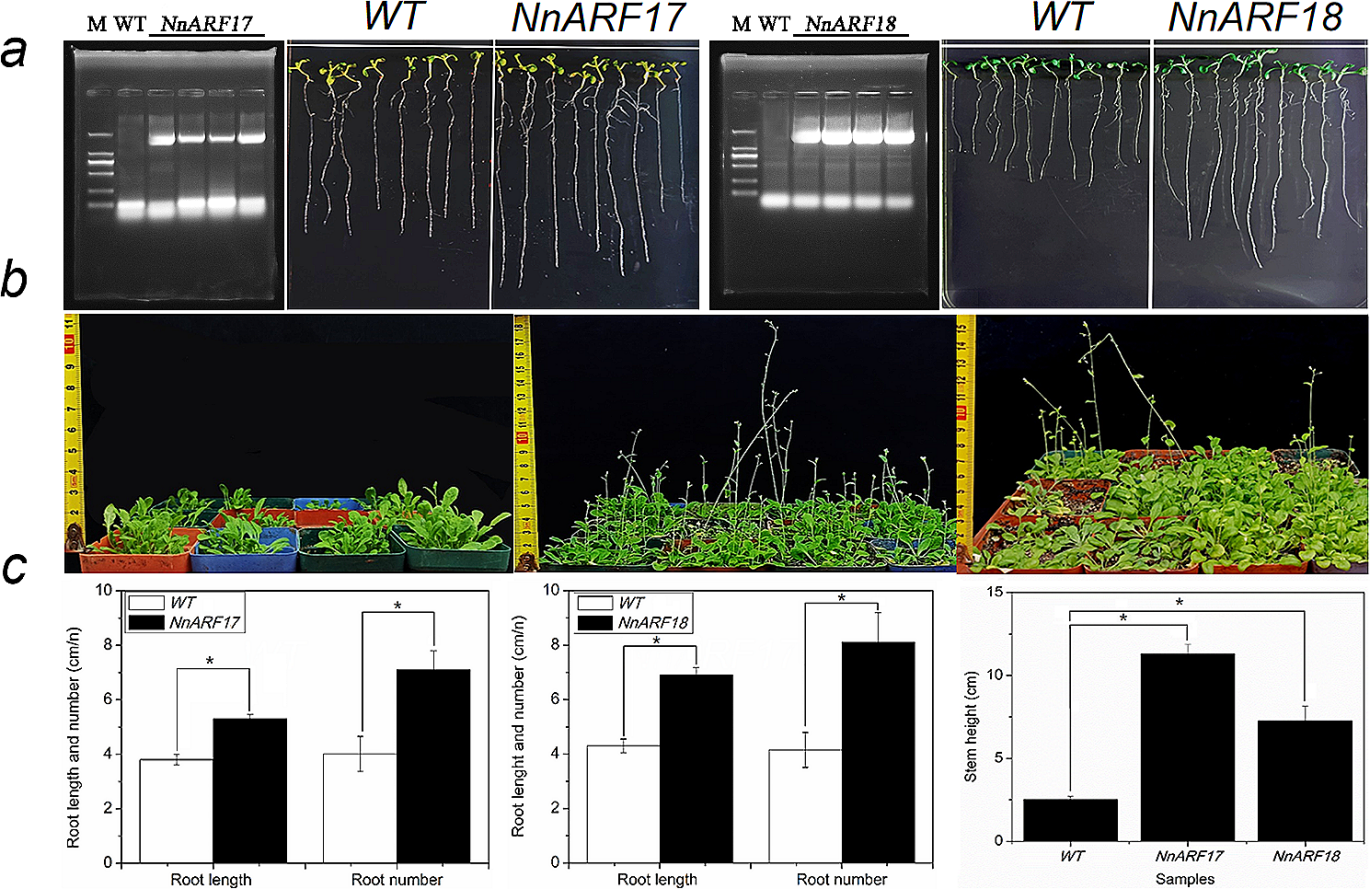
Functional analysis of *NnARF17* and *NnARF18* in transgenic *Arabidopsis* plants. **(a)** Assessment of root development in transgenic plants with constitutive *NnARF17*, *NnARF18* and wild-type plants. **(b)** Effect of *NnARF17* and *NnARF18* on stem growth in transgenic *Arabidopsis* plants. **(c)** Statistic analysis of *NnARF17* and *NnARF18* role on root and stem development. The mean values were calculated from three replicated experiments, and error bars showed standard deviation. Significant differences were determined by Student’s t-test. Statistically significant difference between two samples was presented as * *p* < *0.05*

Successful construction of *pSN1301:NnARF* and *pSN1301:NnARF18* paved the way for investigating the roles of these genes in root formation using transgenic *Arabidopsis* plants. Identification of “positive” plants was achieved through PCR analysis. Notably, transgenic plants expressing *NnARF17* and *NnARF18* exhibited significantly longer roots and a higher abundance of roots compared to wild-type plants, as illustrated in Fig. 5a and c. Furthermore, the influence of *NnARF17* and *NnARF18* extended to stem growth, with *Arabidopsis* plants displaying constitutive expression of these genes showcasing elevated stem heights in comparison to wild-type plants (Fig. 5b and c). These findings underscore the involvement of *NnARF17* and *NnARF18* in diverse biological processes within transgenic *Arabidopsis* plants.

### Analysis of gene expression in transgenic and wild-type *Arabidopsis* plants

**Fig. 6:**
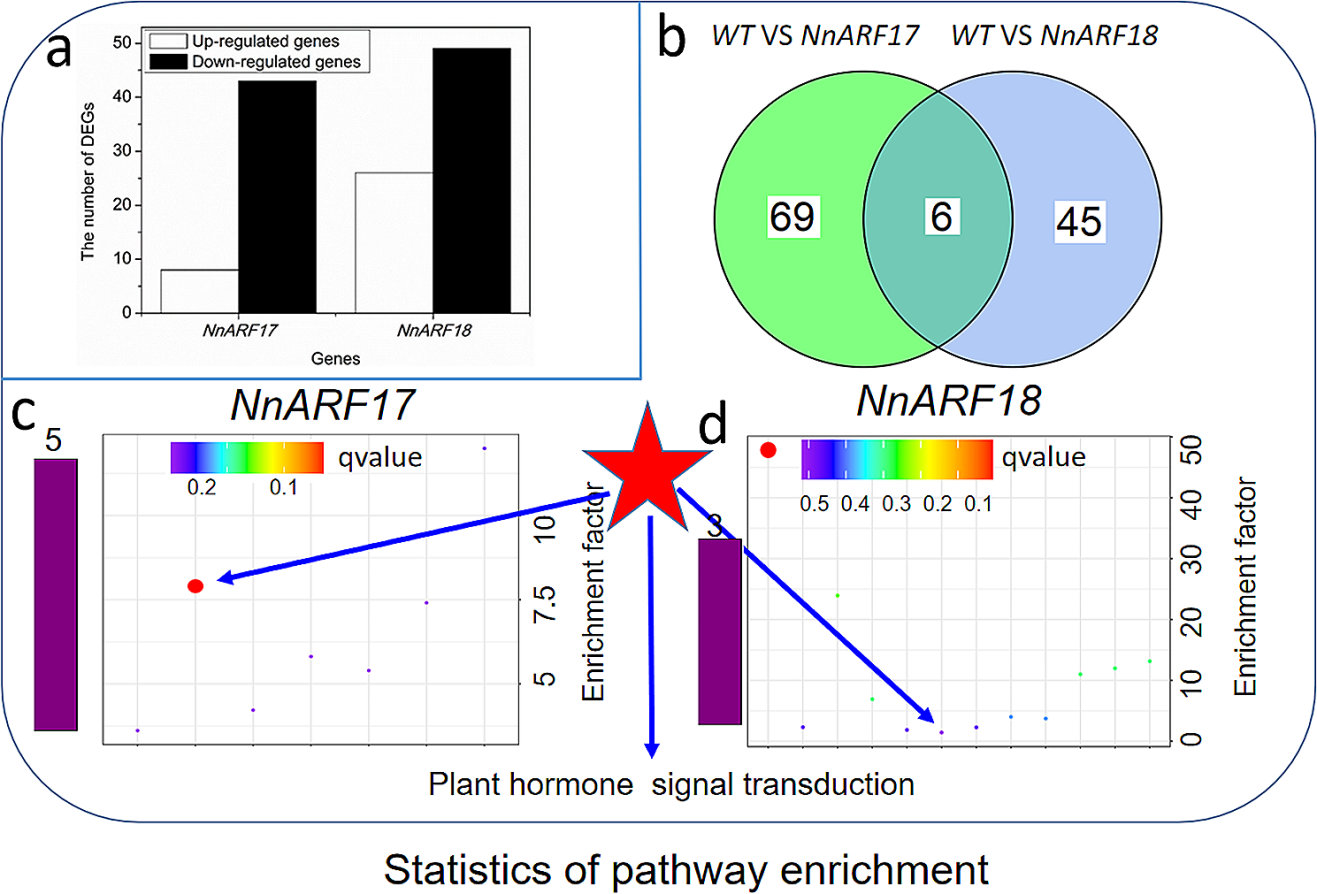
Statistical analysis of DEGs and pathway enrichment in transgenic plants. **(a)** Number of DEGs following overexpression of *NnARF17* and *NnARF18* in *Arabidopsis* plants. **(b)** Distribution of DEGs between *NnARF17* and *NnARF18* transgenic plants. **(c)** Genes involved in plant hormone transduction pathway are counted in transgenic plants expressing *NnARF17* and *NnARF18*.

At the five-to-six-leaf stage, both transgenic and wild-type plants were scrutinized for alterations in gene expression through RNA-seq analysis. The results unveiled distinct transcriptional changes in transgenic plants, with 51 genes, including eight upregulated and 43 downregulated genes, exhibiting modified expression levels in transgenic *NnARF17* plants. Similarly, transgenic NnARF18 plants displayed changes in 75 genes, comprising 26 upregulated and 49 downregulated genes (Fig. 6a).

Comparative analysis revealed that only six genes were implicated in the same biological pathways in both transgenic plants (Fig. 6b). Further exploration of these DEGs pinpointed five genes associated with plant hormone metabolism and signal transduction. Noteworthy candidates included pleiotropic drug resistance 12, SAUR-like auxin-responsive protein family, auxin-responsive GH3 family protein, transcription factor MYC2, ethylene-responsive element binding factor 13, cytochrome P450, ethylene-responsive transcription factor ERF053, and bZIP transcription factor family proteins (Fig. 6c and d; Table 1). Seven genes linked to root formation exhibited altered expression levels (Table 1).

Additionally, scrutiny of stress response-related genes unveiled noteworthy changes. In transgenic *NnARF17* plants, three genes (stress response component-like protein, serine carboxypeptidase-like 50, and NAC domain-containing protein 1) were upregulated, while six genes (stress response component-like protein, plant U-box 29, serine carboxypeptidase-like 50, transcription factor MYB15, transcription factor MYB90, and xyloglucan endotransglucosylase) were downregulated. In transgenic *NnARF18* plants, seven genes (F-box/kelch-repeat protein SKIP6, E3 ubiquitin-protein ligase, glutathione S-transferase U2, NAC domain-containing protein 1, putative POD, fatty acid desaturase 8, and multidrug resistance protein/P-glycoprotein-like) exhibited increased expression levels, while five genes (basic-leucine zipper [bZIP] transcription factor family protein, WRKY DNA-binding protein 45, basic helix-loop-helix [bHLH] DNA-binding superfamily protein, calcium ion-binding protein, and U-box kinase family protein) showed decreased expression levels (Table 2).

**Table 2:** Expression of genes related to stresses response in transgenic *NnARF17* and *NnARF18 Arabidipsis* plants

ID	*NnARF17*	*NnAFR18*		Function annotation
AT4G39840	12.34	------		Stress response component-like protein
AT1G15000	9.54	------		Serine carboxypeptidase-like 50
AT1G01010	1.7	1.62		NAC domain-containing protein 1
AT4G39838	-1.28	------		Stress response component-like protein
AT3G18710	-1.59	------		Plant U-box 29
AT1G15002	-1.76	------		Serine carboxypeptidase-like 50
AT3G23250	-2.92	------		Transcription factor MYB15
AT1G66390	-2.93	------		Transcription factor MYB90
AT4G30280	-3.63	------		Xyloglucan endotransglucosylase
AT2G07475	------	3.60		F-box/kelch-repeat protein SKIP6
AT1G66040	------	2.62		E3 ubiquitin-protein ligase
AT2G29480	------	1.76		Glutathione S-transferase U2
AT2G34060	------	1.18		Putative peroxidase
AT5G05580	------	1.03		Fatty acid desaturase 8
AT4G18050	------	1.00		Multidrug resistance protein/P-glycoprotein-like
AT2G36270	------	-1.01		Basic-leucine zipper (bZIP) transcription factor family protein
AT3G01970	------	-1.25		WRKY DNA-binding protein 45
AT4G28790	------	-1.89		Basic helix-loop-helix (bHLH) DNA-binding superfamily protein
AT2G04755	------	-5.35		Calcium ion-binding protein
AT3G61410	------	-2.24		U-box kinase family protein

Note: “-----” represented no changes of expression

### Determination of IAA, ABA, GA3, and POD contents in transgenic *Arabidopsis* plants

**Fig. 7:**
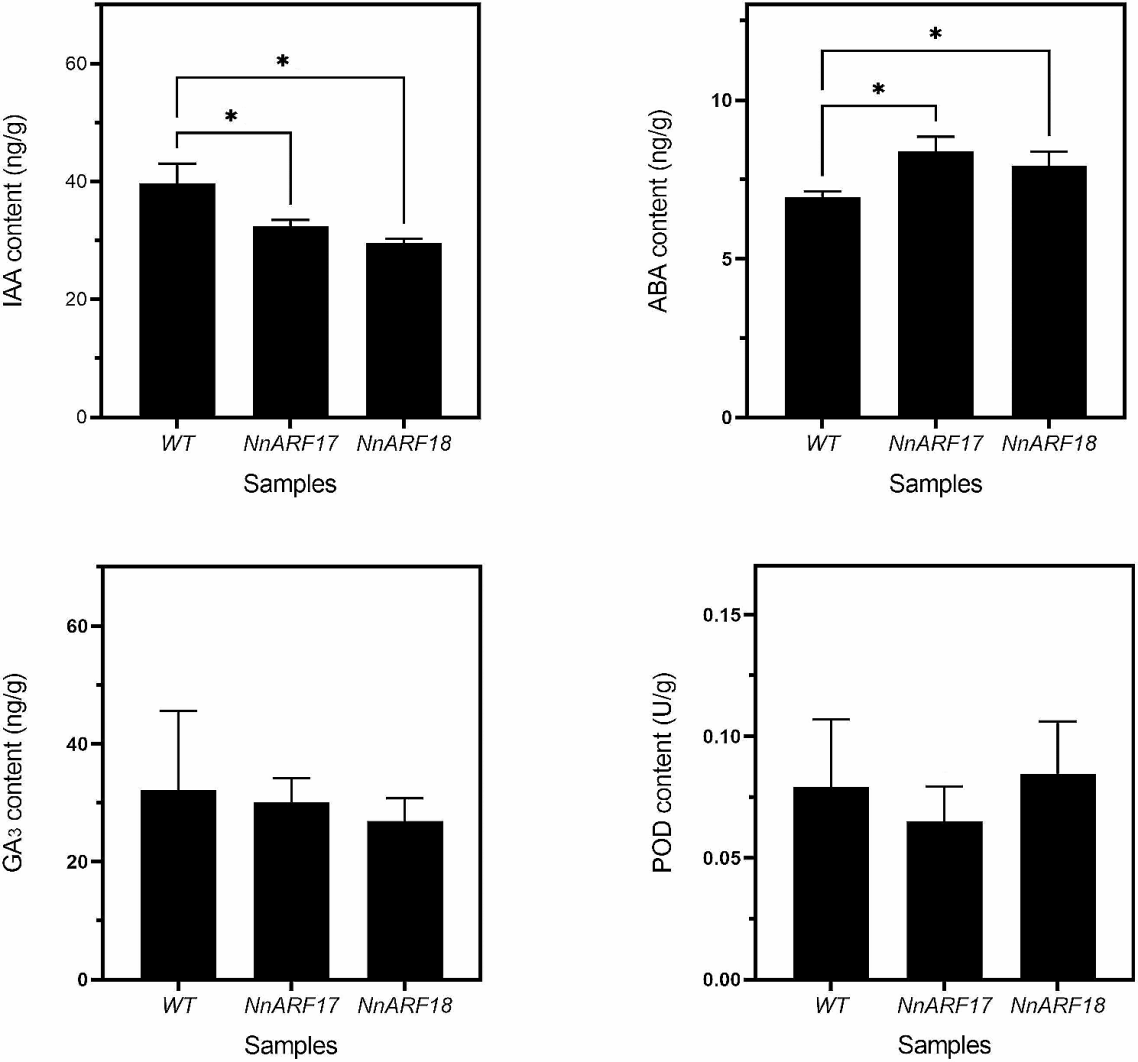
Determination of IAA, GA3, ABA, and POD contents in transgenic plants with *NnARF17* and *NnARF18* and in wild-type plants. For statistical analysis, the data were recorded as means ± SEs of three biological replicates with about twenty seedlings in each experiment. Significant differences were determined by Student’s t-test. Statistically significant difference between two samples was presented as * *p* < *0.05*

Given the pivotal roles of IAA, ABA, GA3, and POD in lotus seedling AR formation, we conducted an analysis of these substances in transgenic *Arabidopsis* plants. The results unveiled a notable decrease in IAA content in both transgenic *NnARF17* and *NnARF18* plants compared to wild-type plants, implying a discernible impact of *NnARF17* and *NnARF18* on IAA metabolism. Furthermore, the ABA content exhibited a significant increase in these transgenic plants, while GA3 and POD content remained unchanged when juxtaposed with wild-type plants (Fig. 7).

To delve deeper, we examined the expression of genes pertinent to IAA metabolism and observed an elevated expression level of pleiotropic drug resistance 12, a member of the ATP-binding cassette superfamily, implicated in auxin transport (Table 1). The precise relationship between the reduced IAA content in transgenic plants and the upregulation of this gene warrants further investigation.

### Adaptation of transgenic *Arabidopsis* plants to waterlogging, drought, and salt stress

**Fig. 8:**
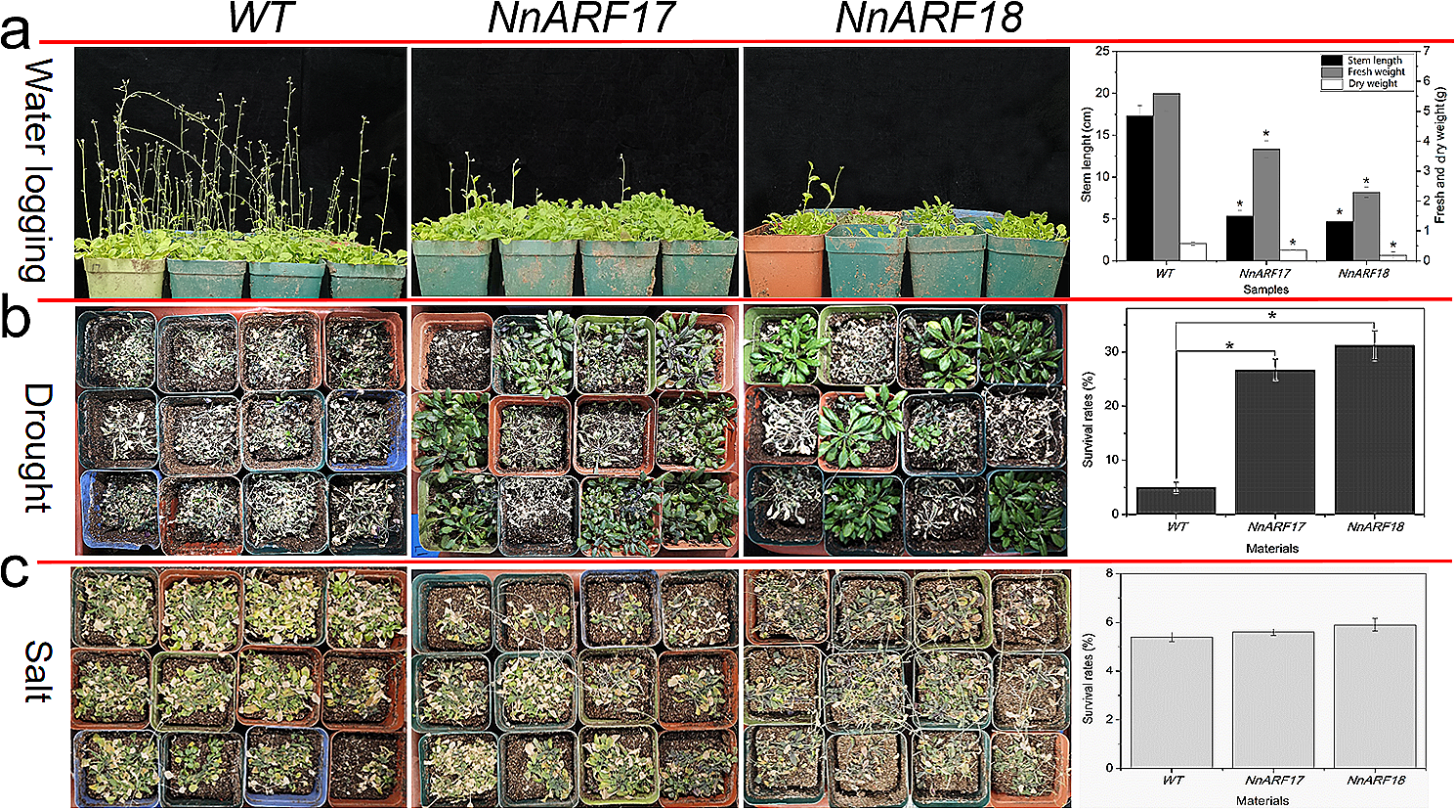
Survival rates of transgenic *NnARF17* and *NnARF18* plants and wild-type plants in response to waterlogging, drought and salt stresses. **(a)** Survival rates of transgenic *NnARF17*, *NnARF18* and wild type *Arabidipsis* plants after waterlogging treatment. **(b)** Survival rates of transgenic *NnARF17*, *NnARF18* plants and wld-type plants after drought treatment. **(c)** Survival rates of *NnARF17*, *NnARF18* transgenic plants and wild type plants after salt treatment. The data were recorded as means ± SEs of three biological replicates with about fifty seedlings in each experiment. Significant differences were determined by Student’s t-test. Statistically significant difference between two samples was presented as * *p* < *0.05*

To unravel the roles of *NnARF17* and *NnARF18* in plant adaptation to adverse conditions, we subjected transgenic plants overexpressing these genes, along with wild-type plants, to waterlogging, drought, and salt stress. The outcomes revealed that transgenic plants exhibited lower survival rates, decreased fresh and dry weights, and shorter stems under waterlogging conditions compared to wild-type plants, indicating reduced adaptation to waterlogging in plants constitutively expressing *NnARF17* and *NnARF18* (Fig. 8a). Interestingly, these transgenic plants displayed enhanced tolerance to drought stress compared to the wild type (Fig. 8b). However, no significant difference was observed between transgenic and wild-type plants under salt stress conditions (Fig. 8c).

In summary, *NnARF17* and *NnARF18*, primarily associated with root formation, exhibit a strong correlation with stress adaptation in transgenic *Arabidopsis*.

### Changes in physiological indices in lotus seedlings after IAA treatment

**Fig. 9:**
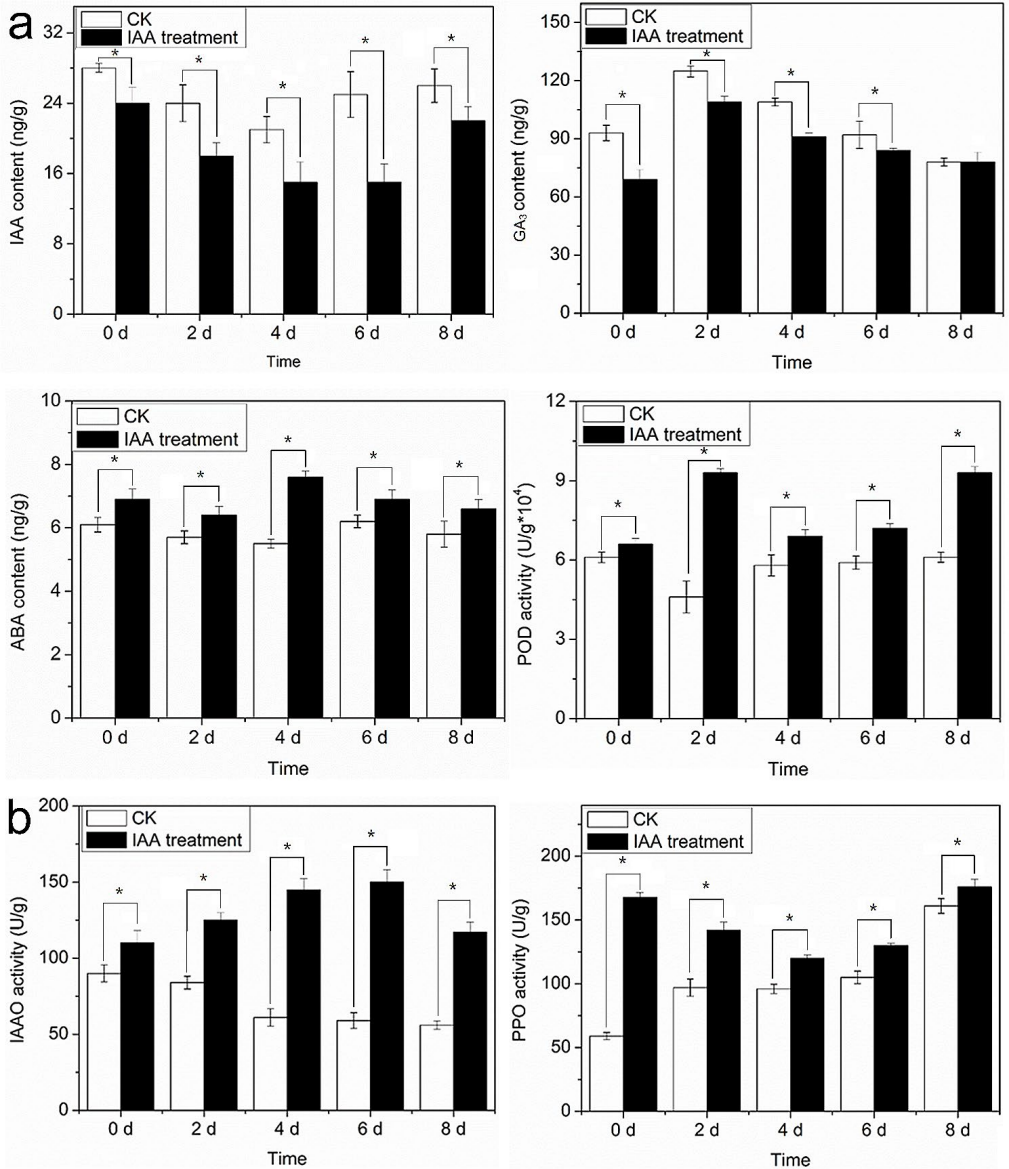
Role of IAA on the development of lotus seedlings. a. Effect of exogenous IAA on the content of IAA, ABA, GA3, POD, PPO and IAAO in lotus seedlings at 0, 2, 4, 6 and 8 d after 10 µM IAA treatment. Each experiment was carried out with three replicates, and the data represents means ± SEs for about 20 seedlings. Significant differences were determined by Student’s t-test. Significant difference between two samples was presented as * *p* < *0.05*

To assess the impact of 10 µM IAA treatment over 2 days on lotus seedlings, we monitored changes in IAA, GA, ABA, IAAO, PPO, and POD content. Notably, the IAA content exhibited a marked decrease from day 0 to day 6 after IAA treatment. The GA3 content initially increased, followed by a significant decrease. The ABA content consistently increased from day 0 to day 8, surpassing the levels observed in control plants. In the context of AR formation, the POD content displayed dynamic changes over the 8-day period: an initial increase from day 0 to 2, followed by a decrease from day 2 to 6, and a subsequent increase on day 8 (Fig. 9a). Further scrutiny of the two enzymes associated with IAA metabolism revealed that the IAAO content exhibited a continuous increase from day 0 to 6, followed by a decrease on day 8. Conversely, the PPO content in treated seedlings decreased from day 0 to 6 and then increased on day 8 (Fig. 9b).

## Discussion

ARs play a vital role in nutrient and water absorption in lotus plants, particularly in light of the underdevelopment of primary roots. These ARs are subject to various factors directly influencing plant growth and environmental adaptation. Our study revealed a significant promotion of AR development in lotus seedlings treated with 10 µM IAA (Fig. 1a). The role of IAA has garnered extensive attention in recent research, with implications in diverse biological processes, such as growth, development, and stress adaptation, despite its low plant content. At the cellular level, auxin, including IAA, regulates plant cell division, elongation, and differentiation [11, 12]. On the organ scale, auxin contributes to the formation of various organs, including roots, buds, leaves, flowers, and fruits [13, 14]. Additionally, auxins play a crucial role in vascular tissue differentiation and plant tropisms, such as gravitropism and phototropism [9, 40]. Specifically, auxin is implicated in root cap development and fosters root formation during AR initiation [15, 16]. Exogenous IAA, as demonstrated in our study, significantly enhances cell division and primordium formation, accelerating the development of AR primordia compared to control plants. This suggests that IAA regulates the initial stage of AR formation (induced stage), prompting further investigation into its impact on the initiation and expression stages of AR development in lotus plants (Fig. 1b).

ARFs play crucial roles as either activators or repressors in plant growth and development, impacting processes like root initiation, apical dominance, tropism, and cellular functions. This extensive family, comprising numerous members [41], has been identified in various species, albeit with varying family sizes [42]. As pivotal transcription factors, ARFs regulate downstream gene expression by binding to auxin response elements in promoters with a consensus sequence [43, 44]. Generally, AUXs/IAAs represent domains within ARFs responsive to auxin stimulation [45]. Furthermore, despite low sequence similarity in the encoding proteins, the B3 domain remains a conserved structure in ARF family members [46, 47].

In our prior study, *NnARF17* and *NnARF18* were found to be upregulated in lotus seedlings following sucrose treatment [34]. In the current investigation, we successfully cloned *NnARF17* and *NnARF18*. Sequence analysis revealed limited homology between the encoded proteins of *NnARF17* and *NnARF18*. However, these proteins shared identical domains (Fig. 2a, b, and c). This phenomenon, also observed in *Salix suchowensis*, where many ARFs exhibit similar expression profiles during different growth stages [48], suggests a connection between this pattern and protein structure. Phylogenetic analysis further supported this hypothesis, placing *NnARF17* and *NnARF18* in distinct groups despite sharing domains and motifs (Fig. 2d). Remarkably, *NnARF17* and *NnARF18* exhibited analogous expression profiles, being induced by IAA, sucrose, and ethephon (Fig. 3). This suggests that despite sequence differences, these genes might not differ significantly in their regulation of plant metabolism.

Auxins play a crucial role in the physiological process of AR formation by orchestrating the expression of downstream genes [49]. In our study, the overexpression of auxin-induced genes, namely, *NnARF 17* and *NnARF18*, was found to significantly promote root formation in transgenic *Arabidopsis* plants (Fig. 4). Numerous key genes are known to be involved in the IAA response, with transport and synthesis playing pivotal roles in AR developmental processes [50, 51]. For instance, ARF6, ARF8, and ARF17 have been identified as contributors to AR development [52].

In the early auxin response, three genes—auxin, *GH3*, and *SAURs*—are rapidly activated by auxin [53]. We observed a downregulation of *GH3* in the transgenic *Arabidopsis* plants (Table 1). GH3, responsible for synthesizing jasmonic acid and IAA, plays a role in jasmonic acid- and salicylic acid-mediated plant defense reactions and photoreactions [53]. Notably, *GH3* mutants have been associated with enhanced development of main and lateral roots [43], suggesting that changes in GH3 expression levels may influence both root development and stress adaptation.

Auxin transport, involving polar, non-polar, and horizontal modes [54], plays a critical role in root formation. Polar transport, occurring from the upper end to the lower end of plant morphology, has extensive physiological roles, including induction of leaf primordium, differentiation of leaf microtubule structures, and stress responses [55–57]. Non-polar transport involves the rapid micro-diffusion of auxins up and down the plant through the phloem [58]. Horizontal transportation, affected by gravity, light, and internal charge distribution, occurs in roots and stem tips [59].

Two types of IAA transporters, influx and efflux carriers, have been identified to date. The AUX1/LAX family, categorized as influx carriers, significantly influences root development by triggering IAA distribution [60, 61]. Our findings indicate that plants with constitutive expression of *NnARF18* exhibit enhanced expression of a gene associated with polar auxin transport. Furthermore, the transcription level of an auxin-responsive gene increases with *NnARF18* overexpression (Fig. 5; Table 1), highlighting the critical role of auxin transport in root formation.

In addition to the recognized positive roles of IAA and ethylene, ABA signaling emerges as a crucial player influencing AR development [22, 62, 63]. Subsequent studies have delved into the nuanced impact of ABA on AR formation, revealing its inhibitory effect primarily during the induction stage of primordium and root elongation stages in softwood cuttings. Interestingly, ABA/IAA is found to be beneficial for the induction of root primordium [23]. ABA exerts its influence on AR formation by augmenting the accumulation and activity of endogenous IAA [64]. Moreover, ABA demonstrates its multifaceted impact by inhibiting the rate of AR growth through modulation of GA biosynthesis and activity, coupled with IAA signaling in rice [24]. Therefore, the ABA / (IAA + GA3) ratio emerges as a valuable indicator of rooting capacity [25].

Intriguingly, our study revealed a novel facet of ABA’s involvement in AR development by enhancing the content of photosynthates, including glucose, sucrose, starch, total sugars, glucose-6-phosphate, fructose-6-phosphate, and glucose-1-phosphate [62]. This aligns with findings in cucumbers, where an intricate interplay between ABA and glucose regulates AR formation, and ABA, in turn, promotes AR development by elevating endogenous ABA accumulation [63].

Our experimental evidence further supports the notion that plants overexpressing *NnARF17* and *NnARF18* exhibit a significant increase in ABA content (Fig. 7), and this observation is corroborated by additional experiments involving lotus seedlings treated with IAA, where ABA content also experiences an upswing (Fig. 8). This suggests a potential positive role of ABA in root formation in both transgenic plants and lotus seedlings. Notably, while there was no discernible change in GA3 content in transgenic *Arabidopsis* plants, a significant increase in GA3 content was observed in lotus seedlings treated with IAA.

IAAO, PPO, and POD emerge as pivotal players intricately linked to the intricate process of AR formation in plants. Among these, POD, a key enzyme in lignin synthesis, plays a crucial role in the initiation and elongation of ARs [65]. Comprising various isoenzymes with diverse physical and chemical properties, POD’s association with plant root formation has been extensively explored, primarily focusing on organogenesis [7, 66, 67]. Our experimental data unveil a significant surge in POD content in seedlings post IAA treatment, underscoring the potential importance of POD in lotus AR formation (Fig. 8).

IAAO and PPO contribute to the regulation of IAA content in plants by degrading IAA, thereby influencing overall plant growth and development. PPO, a plant-exclusive enzyme, catalyzes the condensation of phenols and IAA, forming an “IAA phenolic acid complex” conducive to AR formation. By catalyzing auxin metabolism, PPO promotes the occurrence and development of ARs, with exogenous hormone application amplifying PPO levels and consequently enhancing AR formation [68]. Additionally, studies by Ahkami et al. [69] highlight the influence of auxin on endogenous IAA content through the regulation of *GH3* expression in *Petunia hybrida*. Integrating these insights with our experimental findings (Fig. 7), it becomes evident that the IAA content during root formation is influenced by a complex interplay of multiple factors.

ARFs, known for their roles in stress adaptation, including responses to drought, salt, and low temperatures [70, 71], exhibit their influence in the context of overexpressed *NnARF17* and *NnARF18.* The enhanced survival rates of transgenic *Arabidopsis* plants in response to drought (Fig. 8) coincide with an observed increase in ABA content in plants with constitutive expression of these genes (Fig. 7). ABA, recognized for its extensive responsiveness to various stressors [72, 73], prompts further exploration into whether *NnARF17* and *NnARF18* bolster plant adaptation by modulating ABA content. The dynamic nature of gene expression serves as a strategic response to environmental conditions, especially adverse factors [74, 75]. The observed alterations in the mRNA levels of various genes (Table 2) suggest their potential involvement in the adaptation to drought stress in transgenic *Arabidopsis* plants. Intriguingly, the overexpression of *NnARF17* and *NnARF18*, while enhancing drought tolerance, paradoxically reduced tolerance to waterlogging, drought, and salt stress. This hints at the intricate interplay between gene regulation, IAA content, and stress tolerance, warranting further investigation into the underlying mechanisms.

## Conclusions

This study confirmed the promotive role of IAA in AR formation in lotus seedlings. We identified and assessed two auxin-responsive genes (*ARF17* and *ARF18*) in *Arabidopsis*, revealing low sequence similarity but similar protein structures for *NnARF17* and *NnARF18*. Induction by IAA, ethephon, and sucrose, and expression in various lotus organs characterized these genes. Constitutive expression of *NnARF17* and *NnARF18* positively influenced root and stem development but negatively impacted waterlogging adaptation while increasing drought stress. Transcriptomic analysis using RNA-seq in transgenic *Arabidopsis* plants demonstrated a decrease in IAA content and an increase in ABA content. Additional examination of IAA, ABA, GA3, PPO, IAAO, and POD contents in lotus seedlings treated with exogenous IAA revealed significant increases. In summary, our findings emphasize the indispensable role of IAA in lotus AR formation by regulating downstream responsive genes.

### Electronic supplementary material

Below is the link to the electronic supplementary material.

Supplementary Material 1

Supplementary Material 2

Supplementary Material 3

Supplementary Material 4

## Data Availability

The material of all the experiment was supported by aquatic vegetable Lab of Yangzhou University. The collection of seed complied with local andnational guidelines and permissions of seed were obtained. The detail data has been deposited in NCBI database (Project: PRJNA1047315; WT1, WT 2, WT 3: SRR27065900, SRR27065899, SRR27065898; ARF17-1, ARF17-2, ARF17-3: SRR27065897, SRR27065896, SRR27065895; ARF18-1, ARF18-2, ARF18-3: SRR27065894, SRR27065893, SRR27065892).
