# Supplementary figures and images for: NnARF17 and NnARF18 from lotus promote root formation and modulate stress tolerance in transgenic Arabidopsis thaliana
Source: BMC Plant Biol. 2024 Mar 2;24:163. doi: 10.1186/s12870-024-04852-9 (PMC10908128; doi:10.1186/s12870-024-04852-9)

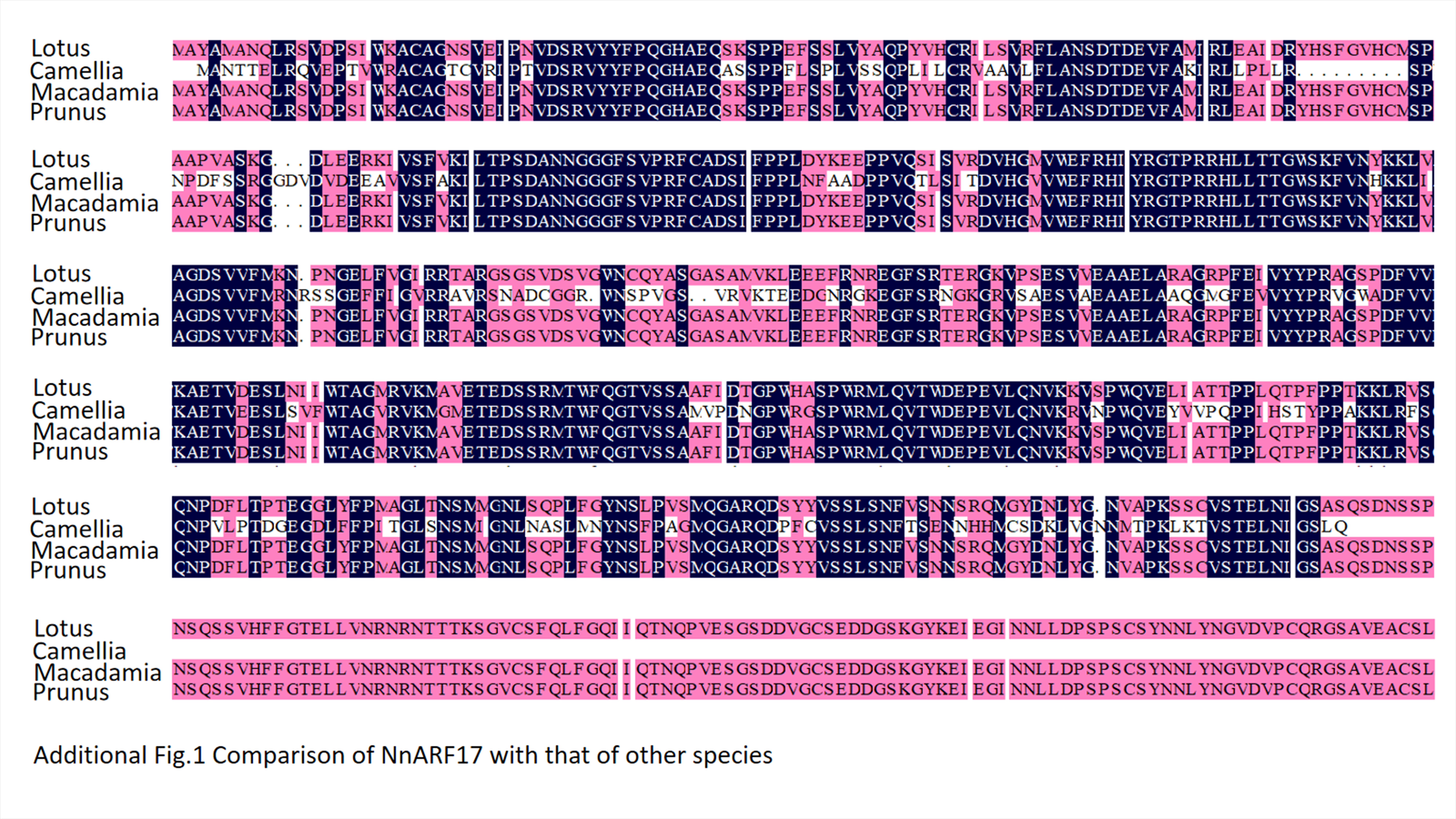

Supplement: Supplementary file 1 — Supplementary Material 1 [file 12870_2024_4852_MOESM1_ESM.tif]

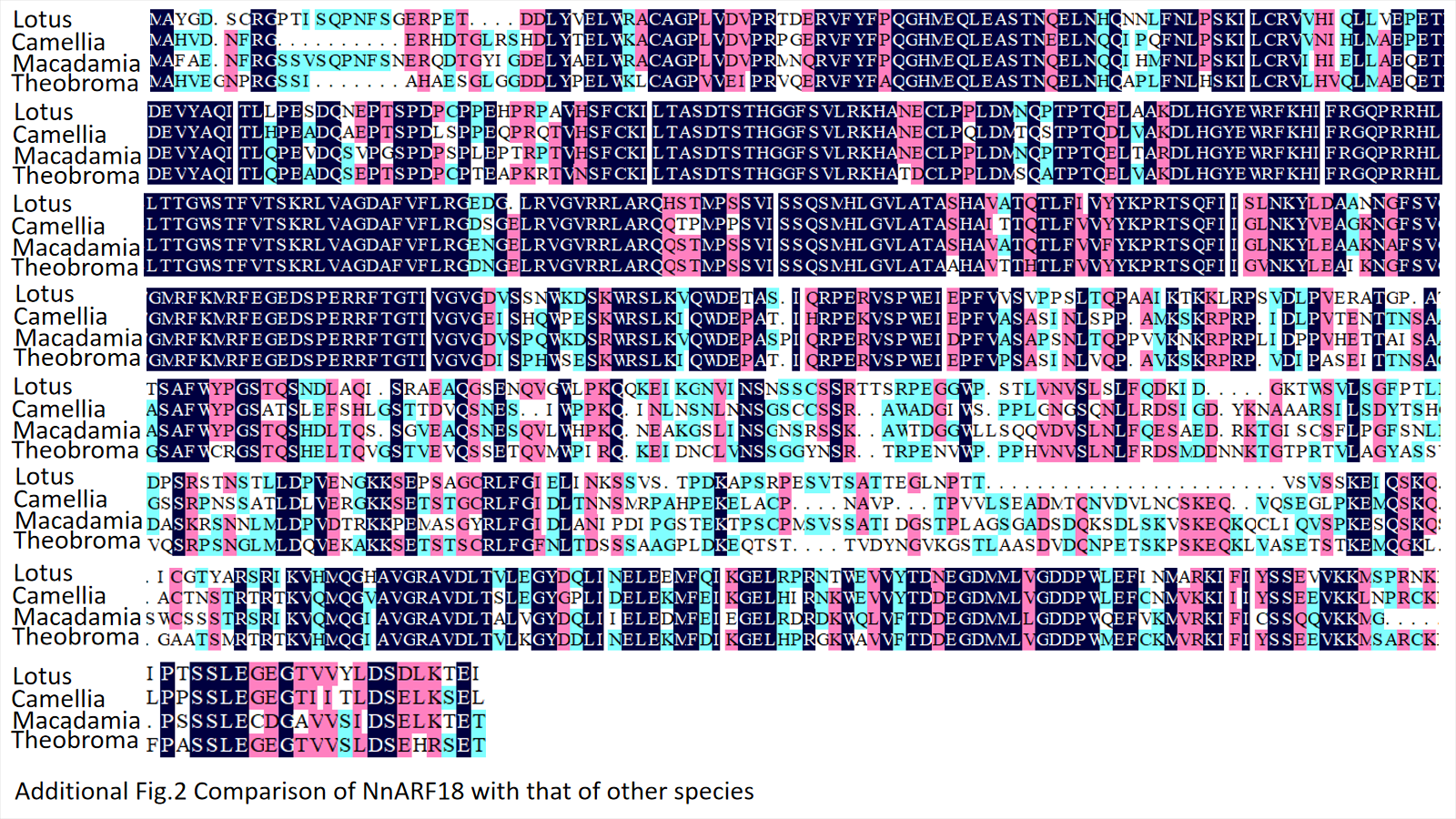

Supplement: Supplementary file 2 — Supplementary Material 2 [file 12870_2024_4852_MOESM2_ESM.tif]
