# Supplementary material for: NnARF17 and NnARF18 from lotus promote root formation and modulate stress tolerance in transgenic Arabidopsis thaliana
Source: BMC Plant Biol. 2024 Mar 2;24:163. doi: 10.1186/s12870-024-04852-9 (PMC10908128; doi:10.1186/s12870-024-04852-9)
Supplement: Supplementary file 4 — Supplementary Material 4 [file 12870_2024_4852_MOESM4_ESM.docx]

Additional table.1. Gene sequences of *NnARF17 and* *NnARF18*.

***NnARF17*:**

ATGGCGTTGCAGAGGGTGAGTGAGCTCCGCAGCCTCGATCCCTACATATGGAAGGCCTGCGCTGGAAACT

CTGTCGAAATCCCCACCGTCGGTTCCACGGTTTACTACTTCCCGCAAGGCCATGCGGAGCAGGCTTCTTC

TTCTCCAGATTTTAGTTCTCTGGCCTGCGTCAGGCACATGGTTCTGTGCAGAATCGTGTCGGTTATGTTC

CTGGCGAATCCTGATACTGATGAGGTTTTTGCAAAAATACGTCTTGAACCGCTGGATCGATCGTTTTCCG

GAATGGTTCGTATGCCGCCGGTTGCAGCGGGATTGTCGCAGGACGGTGACGTTGAAGATTCTGAGGAAAA

GATTTTGTCGTTCTCGAAGATCCTGACGCCAAGCGATGCAAACAATGGAGGTGGGTTTTCGGTTCCCAGG

TTTTGTGCAGATTCAATATTTCCCCCACTGAACTACAAGGCGGAGCCACCAGTACAGACGATTACAGTGA

CAGATGTTCATGGTGTGATGTGGGATTTCCGGCACATTTACCGTGGGACGCCGCGTCGGCATTTGTTGAC

GACTGGGTGGAGTAAGTTCGTTAATCATAAGAAGCTTGTTGCTGGAGATTCAGTGGTTTTTATGAAGAAC

CAAACTGGAGAGCTTTTTGTTGGGGTAAGGCGCACTGCAAGGTCAAGCGGCAGTATGGACTGTGTTCGGT

GGAATTATCATGTCGGGTCTGTGGCTTCTCCCATTGTGAAAATGGAAGAGGGGTTTGGAAGTGGAGAGGG

GTTCTCGAGGAGCAGCAGGGGCAGGGTTCCAGCTGAATCTGTAGTAGAGGCTGCTGAATTAGCAGGAGTA

AACAGGCCTTTTGAGGTTGTTTATTATCCGAGGGCAGGTTCACCGGATTTTGTGGTTAAGGCGGAGGCGG

TAGAGGAATCCCTCAATATAATTTGGATGGTTGGTATGAGGGTTAAAATGGCAGTGGAGACAGAAGATTC

GTCTAGGATGACATGGTTTCAGGGGACTGTTTCGTCAGTTGCAAGTCCAGATCATGGGCCGTGGAGGGGC

TCGTTCTGGCGCATGCTTCAGGTTAACTGGGATGAGCCAGAAGTTTTGCAGAATGTCAAGAGAGTGAGCC

CCTGGCAAGTTGAACTGGTTGCAGCCACACCACCCCTTCAGACCCCATTTCCCCCCACAAAAAAGCTCAG

GGTTCCTCAGAATCCTGAGTTGCTTACTGATGGAGGAGCCCTGTTCTATCCTATGACAGGATTTAATTCA

ATGATGGGAAACTTGAGTCCATCATTGTTTAATTGTAACACTTTTCCTGCTGGCATGCAGGGAGCCAGGC

ATGATCCAATCTGTGTACCCAATTTATCCAATTTCATATCGAGCAATAACCATCAGGTGTTGTGTTCTGA

GAATATCTGTAGCAACAATGTGGCACCAAAATTGAGTTGTATTTCCACCGAATTGAGCATTGGTAGTTCA

TCACAATCAGACAACTCATCACCACATAGCCAGAACAGTGTGCATTTCTTTGGTACTGAAACTTTTGGGA

GCCGAAACAGCAATTTAACAACAAAAGCTACTGTTAGTTCATTCCAATTGTTTGGTAAAATCATCCAGAC

AAAACAGCCTTCTGAGAGTGGGATTGATGACATTGGGTGTATGGAAAATGATGGTGGTAAAGGCTACAAT

GTGACTGAAGCTGTGGCCAATCCACTGGTTCCTGCCTTATCTCACCCTTACAACAAGTTGTATGATGGGG

TTGACATTCAATATCAAGGAGTCTCAGCAGTGGAAGCTTGTTCCTTATG

# *NnARF18:*

ATGGCGTATGGAGATAGCTGCAGAGGCCCCACGATTTCGCAACCGAATTTCTCGGGTGAACGGCCGGAGA

CAGACGATCTCTATGTGGAGCTATGGAGAGCTTGTGCAGGACCTCTCGTCGACGTTCCTCGGACGGACGA

GAGGGTTTTCTACTTCCCTCAAGGTCACATGGAGCAATTGGAAGCATCAACGAACCAGGAGTTGAATCAT

CAGAACAATTTGTTTAATCTTCCTTCAAAGATACTTTGTCGCGTTGTTCACATTCAGTTACTGGTGGAAC

CAGAAACAGATGAAGTTTATGCTCAAATTACCCTACTTCCGGAATCAGATCAAAATGAGCCGACGAGTCC

TGATCCATGCCCTCCTGAACATCCAAGGCCGGCTGTTCATTCCTTCTGTAAGATCTTGACTGCATCTGAT

ACAAGCACACACGGCGGGTTCTCTGTTCTGCGTAAGCATGCCAACGAATGCCTTCCTCCTTTGGACATGA

ACCAGCCTACCCCAACGCAGGAATTGGCCGCTAAGGATCTCCATGGTTATGAGTGGCGATTTAAACACAT

CTTCAGAGGTCAACCACGGAGACATTTGCTTACAACAGGGTGGAGTACTTTTGTTACCTCTAAGCGACTG

GTTGCTGGGGATGCCTTTGTATTCCTTAGAGGGGAGGATGGCCTACGTGTGGGAGTCAGACGTCTTGCTC

GTCAGCATAGCACCATGCCTTCCTCAGTCATCTCCAGCCAGAGCATGCATCTGGGAGTGCTCGCTACTGC

ATCTCATGCAGTAGCTACGCAGACCCTTTTCATTGTCTACTACAAGCCAAGAACAAGTCAATTCATCATA

AGCTTGAACAAATATTTAGATGCTGCTAACAATGGGTTTTCAGTTGGTATGAGATTCAAGATGAGATTTG

AGGGAGAAGATTCTCCCGAGAGAAGGTTTACAGGCACAATTGTTGGGGTTGGAGATGTATCTTCCAATTG

GAAGGATTCTAAGTGGAGATCATTGAAGGTGCAATGGGATGAGACAGCATCCATTCAACGACCAGAGAGG

GTTTCTCCATGGGAGATTGAGCCTTTTGTTGTGTCTGTCCCTCCGAGTCTAACTCAACCAGCAGCCATTA

AGACCAAGAAGCTCCGACCATCAGTAGATCTCCCTGTTGAAAGAGCCACCGGTCCTGCCACCTCAGCATT

TTGGTACCCTGGTTCAACCCAGTCCAATGACCTTGCACAAATAAGTAGAGCTGAAGCACAGGGAAGTGAA

AACCAAGTTGGCTGGCTTCCTAAGCAGCAGAAAGAAATAAAGGGCAATGTCATAAACAGCAACAGCAGCT

GTAGTTCAAGGACGACCAGCAGGCCAGAGGGGGGCTGGCCCTCTACACTCGTGAATGTTTCTTTAAGTCT

GTTTCAGGACAAAATAGATGGCAAAACATGGTCAGTCCTCTCAGGGTTTCCAACCCTGGACCCCTCGAGG

TCAACCAATAGCACGTTGCTTGACCCAGTGGAAAATGGGAAGAAATCTGAGCCATCTGCCGGTTGTCGGT

TGTTTGGGATTGAGTTGATAAATAAATCTAGTGTCTCTACTCCAGACAAGGCACCTTCGCGTCCAGAAAG

TGTAACTAGTGCGACTACTGAAGGGCTTAACCCAACCACTGTGTCTGTGTCATCGAAGGAGATTCAGAGC

AAGCAGATTTGCGGTACTTATGCCAGAAGTCGCATAAAGGTGCATATGCAAGGACATGCAGTTGGTCGGG

CTGTAGACTTGACTGTGTTGGAAGGCTATGATCAGCTTATAAATGAACTGGAGGAGATGTTCCAGATTAA

AGGAGAGCTCCGTCCACGGAATACATGGGAAGTTGTCTACACTGATAACGAAGGAGACATGATGCTTGTG

GGTGACGACCCGTGGCTGGAGTTCATTAACATGGCAAGGAAGATTTTTATCTACTCAAGTGAAGTAGTGA

AGAAGATGAGTCCAAGAAACAAGATTCCCACATCATCTCTGGAGGGTGAAGGGACTGTTGTATACTTGGA

TTCAGACCTAAAGACTGAGATATGA
